# A multi-factorial mathematical model for the selection of electropolishing parameters with a view to reducing the environmental impact

**DOI:** 10.1038/s41598-021-88731-5

**Published:** 2021-05-03

**Authors:** Paweł Lochyński, Sylwia Charazińska, Maciej Karczewski, Edyta Łyczkowska-Widłak

**Affiliations:** 1 Institute of Environmental Engineering, Wrocław University of Environmental and Life Sciences, Grunwaldzki Square 24, 50-363 Wrocław, Poland; 2 Department of Mathematics, Wrocław University of Environmental and Life Sciences, Grunwaldzka 53, 50-357 Wrocław, Poland

**Keywords:** Materials science, Metals and alloys

## Abstract

Electrochemical metal processing is a process that generates harmful pollution. An important goal often disregarded by researchers is not only the achievement of the best possible quality of electropolished surface, but also minimising the load of metal ions in the wastewater generated in the process. The conducted experiments on the electropolishing of stainless steel in laboratory conditions, varied time, temperature and current density conditions, as well as process bath contamination (ranging from 0 to 6% Fe mass) allowed us to develop a multi-factorial mathematical model. This model offers the possibility of being able to select the process parameters recommended for achieving the desired effects. It takes into account such surface quality parameters as roughness and gloss, process duration and current density that determine power consumption, as well as the weight loss of the electropolished element that influence the rate of contamination in processing baths and wastewater. The study presents the composition of a passive film of stainless steel after the electropolishing process at the initial and final stages of the process bath’s exploitation. The results obtained from XPS tests were then correlated with the results of corrosion tests and resistance to pitting corrosion in the environment of 0.1 M NaCl.

## Introduction

Both the steel and metal finishing industries (mechanical and electrochemical treatment of the surface, etc.) have made great strides in terms of development along with a rapid growth in urbanization and infrastructure construction activities. The steel industry has played an important role in the development of human civilization. Associated with its rapid economic expansion, the iron or steel industry is also one of the most energy-consuming and polluting industries^1^. The surface of elements made from stainless steel material is usually subjected to further treatment such as machining, electropolishing, etc. These processes also generate manufacturing waste and have an impact on the environment^2^. In recent years, heavy metals in wastewater have become a major problem for the environment due to the high risk for the ecosystem and human health, even at very low concentration^3,4^. The electroplating industry is a source of chromium, nickel and iron ions which are dangerous for both humans and other living organisms. Cr(III) is not such a strong oxidizer as Cr(VI) and consequently exposure is less harmful to humans, but under certain conditions trivalent chromium compounds such as Cr_2_O_3_ and Cr(OH)_3_ may oxidize to hexavalent chromium^5^. Excessively high levels of nickel can be potentially hazardous and toxic to aquatic organisms. The accumulation of nickel in the environment may represent a serious hazard to human health. The known adverse effects of nickel on human health include skin allergies, lung fibrosis, variable degrees of kidney disease, cardiovascular system poisoning, and stimulation of neoplastic transformation^6,7^.

Anodic dissolution of stainless steel increases the concentration of heavy metal ions like iron, chromium and nickel in the process bath and, eventually, introduces the pollutants to the environment. As a result of the high viscosity of the electrolyte, the wastewater generated during the rinsing of the electropolished workpieces is acidic, along with an increasing heavy metal ions content. The main sources of wastewaters and pollutants in the electroplating industry are cleaning and rinsing^8^. Numerous methods of treatment have been suggested for the removal of heavy metals ions from wastewater, including chemical precipitation, reverse osmosis, ion exchange, foam formation, etc.^9,10.^ The main disadvantage of most of the above processes is the fact that they can produce large amounts of sludge and there is a small possibility of metal recovery. The use of plants and plant-based organic materials for the removal of heavy metals has already been discussed in the literature as non-conventional adsorbents^11–13^.

Apart from searching for new methods to neutralise wastewater generated by the steel electropolishing process, it is also important to attempt to reduce the load of contaminants that emerge in the process. The minimisation and reduction of the concentration of metals that emerge during the process should lead to their reduced load in the generated wastewater, such that further neutralisation becomes easier and more efficient. Many researchers seem to omit this issue, as opposed to the reduction of process costs, shortening the duration and improving the surface quality of the electropolished elements^14^. Thus, the issue of limiting contamination while at the same time optimising the electropolishing process requires further research and discussion.

The electropolishing (EP) process allows for the smoothening, glossing, and improving of the appearance and corrosion resistance of the elemental surface. The EP process removes the microstresses in the superficial layer caused by processing and restores the uniform micro-hardness of the native material. It also enables polishing surfaces and places that are inaccessible for mechanical polishing methods. The most noticeable advantage of the electropolishing process is improvement in the surface appearance. As a result of the minimisation or complete removal of stains and small scratches, electropolishing makes it possible to highlight the surface gloss. Stainless steel corrosion can also occur much faster than expected in certain types of environment in pharmaceutical manufacturing^15^ and in other industries^16,17^. Insidious corrosion, or corrosion that occurs in localized environments rather than generalized corrosion, can be extremely destructive because it is difficult to detect. Pitting and crevice corrosion are examples of this phenomenon. Additionally, the electropolishing process may improve the surface resistance of stainless steel elements to corrosion^18^ which is especially true for elements used in an adverse environment. The obtained final state of the electropolished element’s surface also depends on the initial preparation of the surface before initiating the EP process—number of crevices, mechanical processing, and degreasing.

The literature provides numerous analyses of the electropolishing process. However, most of those studies used high current densities (> 10 A/dm^2^)^19,20^ or even such high current density as 50–1000 A/dm^2^^21,22^. Unfortunately, in industrial conditions, such high current density is impossible to obtain due to the large surface area of the electropolished elements. Few laboratory experiments were conducted for low current densities (< 10 A/dm^2^). Awad^23^ presented the results of electropolishing of 304 steel at a current density ranging from 1.25 to 25.5 A/dm^2^. Positive surface gloss effects of G = 1750, with a weight loss of 27 mg/cm^2^, were obtained at a current density of 3.75 A/dm^2^. There are also works of adaptation to use deep eutectic solvents in the electropolishing process of 304 and 316 stainless steels, although these aplications are yet to be used in industrial practice^24,25^.

Researchers often use mathematical models to describe technological processes and to predict further research results^26,27^. Monitoring test results with the use of linear or non-linear regression may support the control of technological processes in galvanising plants. Due to the economic aspect and industrial applications of the electropolishing process, the appropriate development of surface roughness and gloss after electrochemical treatment is pivotal where selection of the most favourable conditions is concerned^28^.

The aim of the study is to determine the influence of such electropolishing process parameters as time, temperature, current density and bath contamination on the obtained effects. The tests presented in this paper were conducted at current densities of 4 and 8 A/dm^2^ and temperatures of 35 °C, 45 °C, and 55 °C, which correspond to the values applied in the industry. The expected effect of electropolishing was to improve surface quality while maintaining a minimum level of weight loss and electricity consumption.

## Experimental procedures

### Experimental circuit

The electropolishing equipment consisted of the following elements: laboratory power supply unit KP-131 (KP-Elektronika), electric charge counter (KP-Elektronika), EUROSTAR 60 control mechanical mixer (IKA), and water bath (Pilot ONE Huber CC-K12). For the purposes of the experiment, a circuit was made (constructed from a 1.5 mm thick copper sheet) that ensured current flow by using copper bolted connections. During the electropolishing process, the anode was placed centrally, while two cathodes were located 20 mm from the central axis. The EP process was carried out in a glass vessel with a volume of 1000 cm^3^. EP process conditions were as follows: current density 4 and 8 A/cm^2^, time from 5 to 45 min, speed range 50 rev/min, bath temperature 35, 45, 55 ± 1 °C.

### Materials

Tests were conducted on cold-rolled 1.5 mm thick AISI 304 stainless steel 2B surface finish. The chemical material composition was the following (wt%): 0.028% C, 0.37% Si, 0.066% N, 1.39% Mn, 0.034% P, 0.002% S, 18.23% Cr, 8.02% Ni, and balance Fe. Samples 30 mm wide and 90 mm long with a hole of a diameter of 12 mm located 5 mm away from the upper edge were used for the experiments. In order to ensure similar initial conditions before further processing, samples were selected based on roughness and gloss measurements. Raw samples were characterised by an average roughness in the range of 160 ± 10 nm and gloss 200 ± 50 GU. The upper part of the samples was protected with a PTFE tape, and the working exposure surface was 0.4 dm^2^. Before surface treatment, the samples were degreased in an EMAG Emmi 60 HC ultrasound washer in acetone for 20 min, and weighed on Mettler Toledo XS 204 Analytical Balance. After the electropolishing process, samples were rinsed in distilled water and weighed again. Corrosion tests were conducted on 1.5 mm thick AISI 304 stainless steel samples with 12 mm in diameter.

### Chemicals

Tests were conducted by means of two baths: Solution A was a phosphate and sulphate bath, and Solution B was a phosphate and sulphate bath with the addition of triethanolamine. To prepare 1 L of the bath, 96% analytical grade sulphuric (VI) acid, 85% analytical grade orthophosphoric (V) acid and analytical grade triethanolamine, which accounted for 3% of the whole solution’s mass, were used. During the electropolishing process, these baths were gradually contaminated as a result of the anodic dissolution of chromium and nickel steel at a range of 0–6% mass Fe.

### Roughness tests

Mitutoyo Surftest SJ-301 was used for surface roughness tests. The surface roughness of the samples was measured using the profile method. For each sample, six measurements were conducted at the central axis, parallel to the upper edge of the sample.

### Gloss tests

The Rhopoint IQ goniophotometer was used for gloss tests. The device enables gloss finish measurements at angles of 20/60/85°. Following the manufacturer’s recommendations, 20° angle gloss for high gloss finishes was used as the main measurement parameter. Six measurements from one side were taken for each sample.

### XPS surface analyses

The XPS analyses were performed using a PHI 5000 VersaProbe spectrometer (ULVAC-PHI) based on the procedures developed at the Institute of Physical Chemistry, Polish Academy of Sciences^29,30^. The spectrometer was equipped with a monochromatic Al Kα radiation (hv = 1486.6 eV) X-ray source operating at 100 µm spot size, 25 W, and 15 kV. The high-resolution (HR) XPS spectra were performed with the hemispherical analyzer. The pass energy was 117.4 and the energy step size was 0.1 eV. The X-ray beam was incident at the sample surface at an angle of 45° with respect to the surface normal, and the analyzer axis was located at 45° with respect to the surface. The Shirley background and a Gaussian peak shape with 30% Lorentzian character were used to conduct deconvolution of all HR XPS spectra. Characteristic sensitivity factors for X-ray monochromatic source were used to estimate the quantitative chemical composition of the investigated samples (MultiPak or Thermo database). The thickness of the passive film was determined using an Ar^+^ ion beam, energy 500 V, time 0.5 min at a rate of 1.37 nm/min, which was calibrated by measuring the sputtering depth per minute in a standard specimen (SiO_2_).

### Corrosion tests

The corrosion tests were taken in 0.1 M NaCl solution at a temperature of 25 °C, applying a thermostated glass cell with a volume of 1000 cm^3^. Tests were performed with the use of Solartron SI 1287 potentiostat manufactured by AMETEK. Potentiodynamic tests were conducted in a tri-electrode setup. A saturated calomel electrode (SCE) as a reference electrode, a platinum plate that served as an auxiliary electrode, and a steel working electrode were all used. A potential sweep rate of 1 mV/s was applied for the potentiodynamic experiments. The surface area of the working 304 stainless steel electrode samples exposed to the electrolyte was 1 cm^2^. Potentiodynamic measurements were conducted after the open circuit potential test. The obtained data from the corrosion tests samples were analyzed using the dedicated CorrView software.

### ICP tests

The collected EP bath samples were investigated for their metal contents using the ICP-OES method. ICP-OES (inductively coupled plasma-optical emission spectroscopy) tests were conducted using the Thermo Scientific iCAP 7000 Series ICP-OES apparatus. Qtegra Intelligent Scientific Data Solution software was used for data processing.

### Statistical methods

Prediction of gloss and roughness measurements using several variants of regression analysis was performed. Variables included in the analysis were the length of process, temperature, current density, and bath contamination.

As far as roughness analysis was concerned, all possible configurations of interactions between parameters were tested for several types of models, including the square model, third- and fourth-order models, and the logistic model. Due to the fact that the authors had noticed certain local anomalies that did not correspond to the specificity of the analysed process, and in order to eliminate the overfitting phenomenon that could have reduced the effectiveness of the model were actual data to be used, the analysis of roughness was performed using the linear model with a squared component of time. Its high variability relative to the number of time points rendered a higher order of function as nonviable. The model was constructed for a level of pollutants at up to 4% given that the number of defects affecting roughness was very high. Its fit was assessed using R^2^ and the Akaike information criterion.

To analyse the gloss levels, the authors used a nonlinear logistic growth model which is commonly used in modelling population growth as given by the Eq. (1). Its S-like curve and upper limit fitted the overall behaviour of data points:

1 $$f\left( x \right) = \frac{\alpha }{{1 + e^{{ - \left( {\beta + \mathop \sum \nolimits_{i = 1}^{n} \varphi_{i} *x_{i} } \right)}} }}$$

To account for a logistic model starting in 0 gloss units, the outcome of the model needs to be shifted by 200 gloss units. In the case of Solution A and because of huge differences in behaviour based on the contamination level, we opted to split the model into two separate functions, depending on whether the contamination levels were lower than 4% or higher than 4% (or equal). Using a single model yielded a significantly lower fit. As for Solution B, there was no significant drawback for using a single model. The literature suggests that using R^2^ to assess the fit of a nonlinear model is incorrect, since the total variance of a non-linear model is not equal to the sum of explained variance and error variance. The evaluation of R^2^ is an inadequate measure for nonlinear models in pharmacological and biochemical research. Because of our model’s nonlinear nature, its fit was assessed based on the RMSE (root-mean-squared-error) of the fitted model.

## Results and discussion

### Roughness and gloss measurements

**Figure 1:**
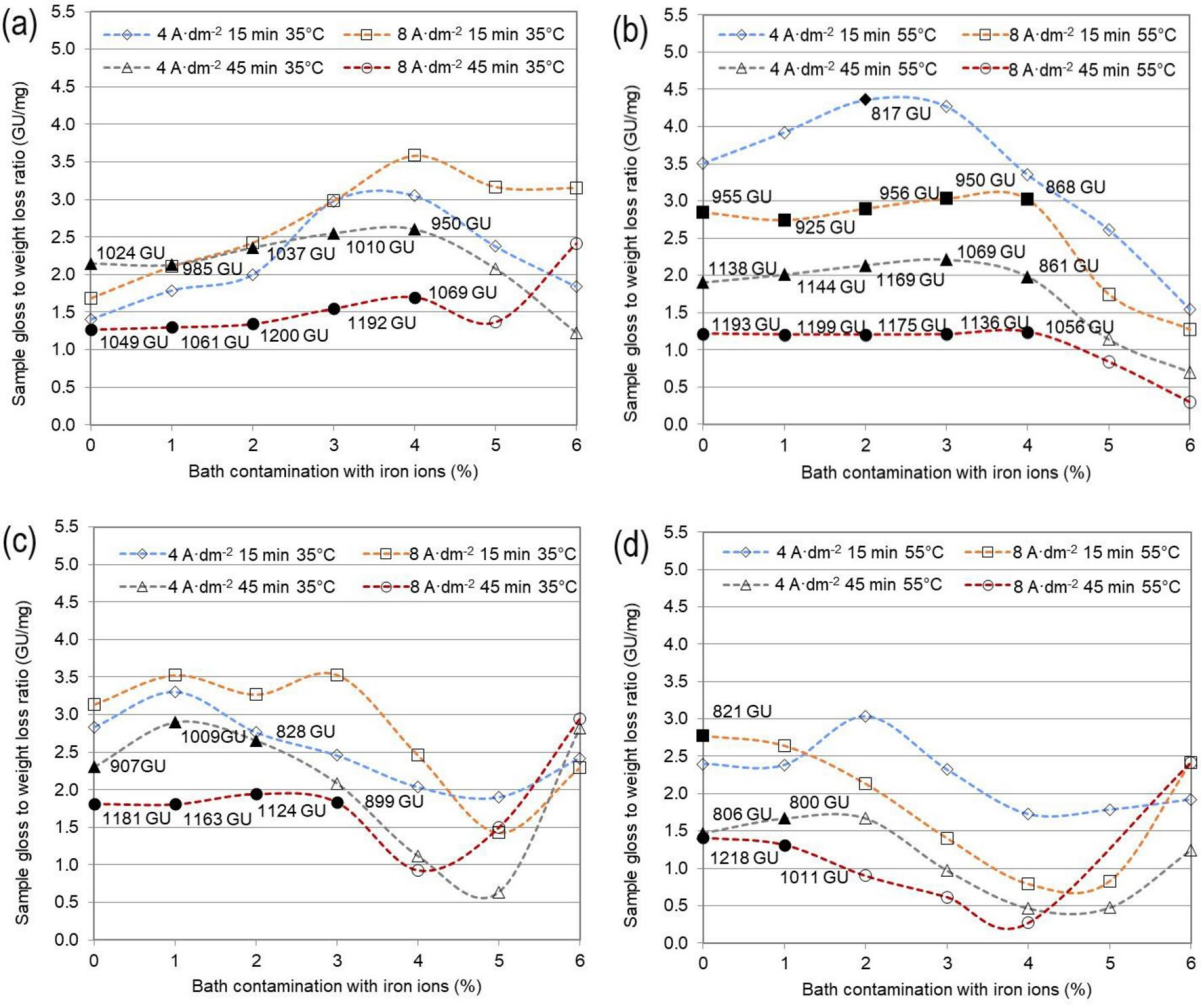
Change in the ratio of sample gloss after EP to the weight loss resulting from EP in a phosphate and sulphate bath for current densities 4 A/dm^2^ and 8 A/dm^2^ as a function of the changing contamination with iron ions ranging from 0 to 6% mass for process temperatures: (**a**) Solution A, T = 35 °C; (**b**) Solution A, T = 55 °C; (**c**) Solution B, T = 35 °C; (**d**) Solution B, T = 55 °C. *Black markers indicate the points whose gloss after EP exceeded 800 GU.

Due to the large surface area of the electropolished elements, it is often impossible to achieve high current density values in industrial conditions. Additionally, it may lead to the overheating of elements while surface defects may emerge in areas of very intense oxygen emission on the anode. At this stage of studies on the exploitation of processing baths, the authors decided to limit the analysed current densities to 4 and 8 A/dm^2^. This selection was based on the fact that those process parameters are used in industrial conditions. An issue that is quite often disregarded in studies on electropolishing is the change in weight loss and gloss of the electropolished elements with the increasing time of operation of the bath. The values of the obtained surface gloss (GU) in relation to weight loss (mg) are presented in Fig. 1. Such a presentation should enable a relatively simple and swift comparison of the results for samples of the same exposed surface area. As presented in the diagrams, the obtained results fall within a range of 0–4.5 GU/mg. Each of the diagrams shows 28 measurement points for the current density of 4 and 8 A/dm^2^. Values, for which the surface gloss exceeded 800 GU, are marked by filled markers. In most cases, the gloss obtained in Solution A at 35 °C, both at 4 A/dm^2^ and 8 A/dm^2^, exceeded 1000 GU, while the contamination with iron ions ranged from 0 to 4. At a 3–4% contamination, a local extreme is noticeable as well as the best gloss to the weight loss ratio for 8 A/dm^2^, 15 min, at a temperature of 35 °C, and a 4% contamination with iron ions. However, a surface gloss exceeding 800 GU was not obtained for this result. At a temperature of 55 °C the relation is similar, but a 5% contamination of the bath results in a deteriorated surface quality. For 8 A/dm^2^, 55 °C, and 15 min, within a contamination range of 0–4%, the sample’s value of the gloss to weight loss ratio is 1.25 GU/mg. A high level of this ratio is obtained at 4 A/dm^2^, 15 min, 55 °C, and a contamination of 2%. Solution B lost its quality fairly quickly. The results at lower temperatures were comparable, whereas for higher temperatures they were several times worse.

**Figure 2:**
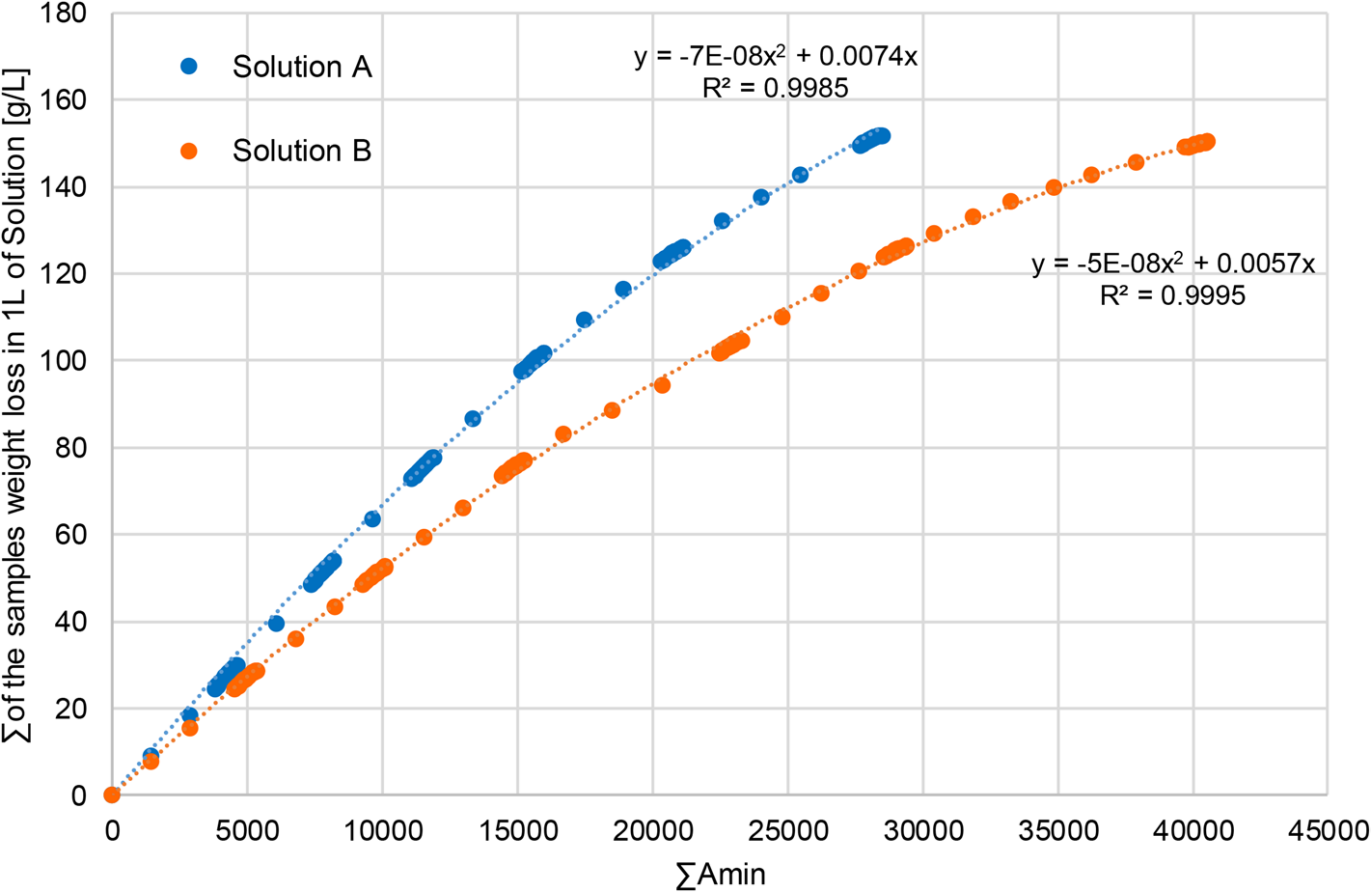
Change in the weight loss of samples as a function of the load expressed in A min for Solution A and Solution B.

The experimental data served as a basis for generating diagrams (Fig. 2) that represent the relation between the weight loss of 304 steel to the electric load of the analysed baths. The increasing bath contamination is accompanied by a decreasing weight loss. In the initial phase of the electropolishing process, the weight loss increases according to Faraday’s equation, i.e. proportionally. As the electric load Amin increases, the bath composition changes while the values of weight loss begin to diverge from those that may be calculated from a linear proportion. The application of organic additives to process baths allows for reducing weight losses in comparison to baths without any additives^31^. The weight loss after the electropolishing process in Solution B, which contained triethanolamine, was lower than that in Solution A which did not contain this additive. With the increasing time of bath exploitation, this difference became higher. In order to achieve the same final total level of weight loss of samples for both baths, Solution B had to be operated for over 1200 A min longer, which accounted for nearly 30% of the total operation time. The weight loss analyses in laboratory conditions produced very similar results to those obtained in industrial conditions using the same process bath^32^. Thus, it is possible to predict the expected weight loss in industrial conditions, even for electropolished elements of a much larger surface area.

### XPS analysis

The chemical composition of the sample surface was analysed with the use of XPS tests. Those studies describe the composition of passive films obtained after electropolishing in baths with various amounts of contaminants—0% and 6% Fe by mass. The chemical compositions (at%) of the analysed samples’ surface before and after sputtering with Ar^+^ ions are presented, respectively, in Tables 1 and 2. The forms (Cr^0^, Ni^0^, Fe^0^) were not taken into account in calculations of the passive film content.

**Table 1:** Analysis of samples before the process of sputtering the surface with Ar^+^ ions, based on high-resolution XPS spectra.

Energy bonds	Chemical composition	A_Fe0%_	A_Fe6%_	B_Fe0%_
707.6–710.2	Fe–O (Fe_3_O_4_), Fe_3_C	5.42	5.29	8.93
708.9–709.4	Fe–O (FeO)	3.80	12.82	12.33
710.4–710.8	Fe–O (Fe_2_O_3_)	17.25	13.39	–
711.8–712.0	Fe–S (FeSO_4_)	2.93	6.52	3.65
711.9–713.2	Fe–P (FePO_4_)	–	3.38	6.54
575.1–575.4	Cr–N (CrN)	6.72	–	21.01
576.1–577	Cr–O (Cr_2_O_3_)	49.46	53.09	29.68
577.8–579.6	Cr–O (CrPO_4_, CrO_3_, Cr(OH)_3_)	14.42	5.51	17.86

**Table 2:** Analysis of samples after the process of sputtering the surface with Ar^+^ ions, based on high-resolution XPS spectra.

Energy bonds	Chemical composition	A_Fe0%_	A_Fe6%_	B_Fe0%_
853.6–853.8	Ni–O (NiO)	1.22	2.45	0.92
854.6–855.6	Ni–O (Ni_x_O_y_)	0.24	0.43	0.39
707.3–707.9	Fe–O (Fe_3_O_4_), Fe–C (Fe_3_C)	17.56	12.27	24.24
709.1–709.2	Fe–O (FeO)	12.32	11.69	11.73
710.6–711.3	Fe–O (Fe_2_O_3_)	5.49	5.20	6.06
574.5–575.2	Cr–N (CrN)	4.39	–	8.04
576.0–576.6	Cr–O (Cr_2_O_3_)	49.63	45.31	31.49
577.4–577.7	Cr–O (CrPO_4_, Cr_x_O_y_)	–	15.58	17.13
578.6–578.8	Cr–O (CrO_3_)	9.15	7.07	–

Table 1 presents the XPS results of the chemical composition of the surface before sputtering with Ar^+^ ions. CrN has energy bonds between 575.1 and 575.4 eV, while the range of energy bond CrN as presented in the literature is 575.6 eV^33^. The formation of PO_4_^2−^, SO_4_^2−^ with Cr(III) and Fe (II) species at the electropolished surface samples is possible^34^. The phosphorus in the metallurgical composition of 304 stainless steel may be oxidized at the surface during the electropolishing process^18^. The deconvolutions conducted for O1s, P2p, and S2p confirm the presence of phosphates and sulphates on the surface of the electropolished samples.

**Figure 3:**
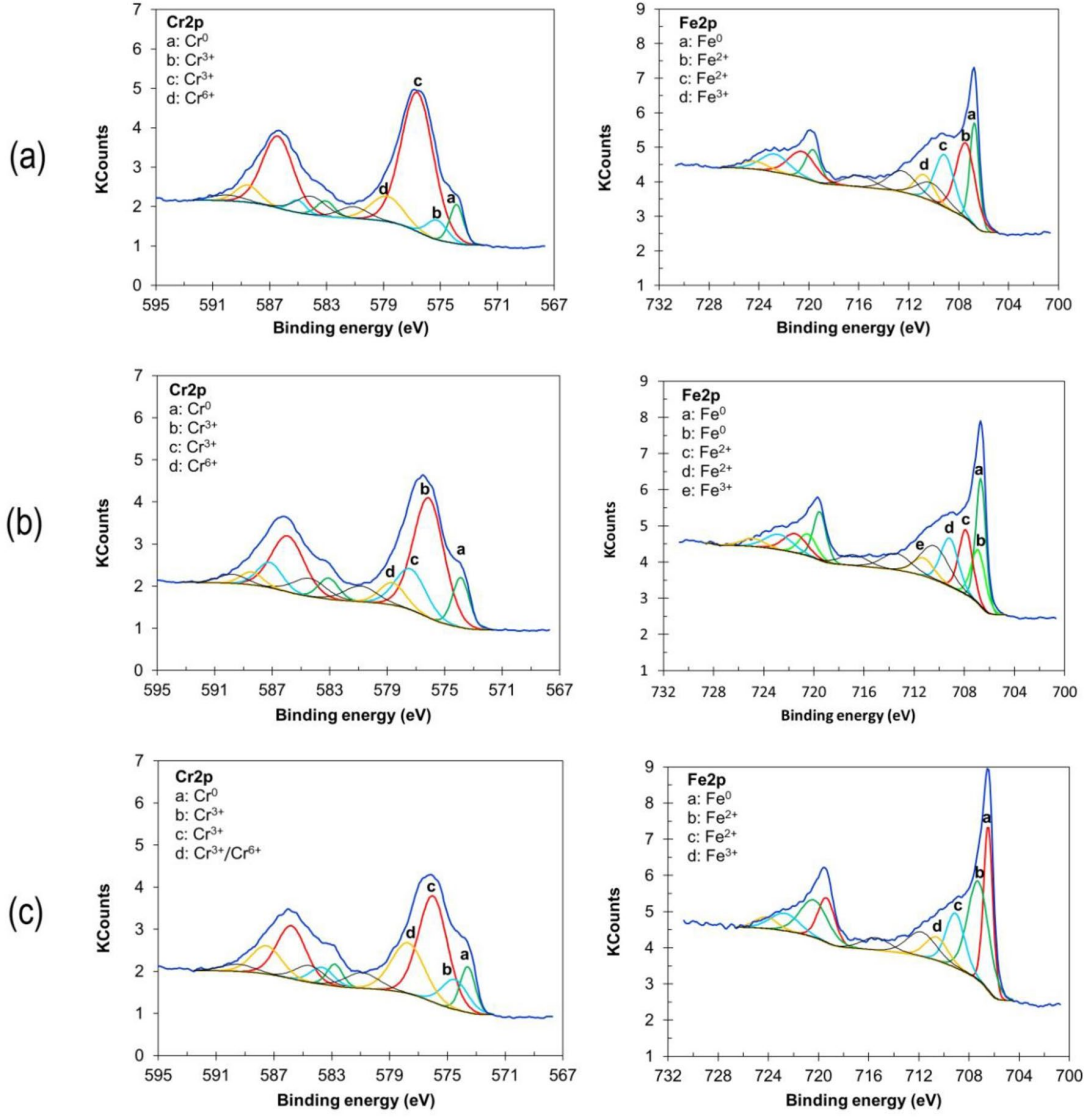
Cr2p and Fe2p deconvolutions of the analysed samples after the electropolishing process: (**a**) solution A_Fe0%_ sample after sputtering for 1.5 min with Ar^+^, (**b**) solution A_Fe6%_ sample after sputtering for 1.5 min Ar^+^, (**c**) solution B_Fe0%_ sample after sputtering for 1.0 min Ar^+^.

Figure 3 presents the resolution Fe 2p3/2 spectra for 304 stainless steel samples after electrochemical polishing and sputtering with Ar^+^. The deconvolutions corresponding to Fe^2+^ oxides (FeO) are between 709.1 and 709.2 eV and for Fe^3+^ oxides (Fe_2_O_3_) between 710.6 and 711.3 eV (Table 2). Other studies have outlined iron XPS peaks in the range of Fe^2+^ oxides (FeO) between 709 and 709.6, Fe^3+^ oxides (Fe_2_O_3_) between 710.5 and 711.5 eV^35,36^, and FeOOH between 711.0 and 711.8 eV^37,38^.

As shown in Fig. 3, the chromium component is characterised by two 2p peaks: Cr2p1/2 and Cr2p3/2. Chromium is present as Cr^3+^ (Cr_2_O_3_) at 576.0–576.7 eV, for Cr^6+^ (CrO_3_) at 578.6–578.9 eV (Tab. 2). Other studies have suggested 576.0–576.8 eV for Cr^3+^ oxide (Cr_2_O_3_)^38,39^, hydroxide (Cr(OH)_3_) at 577.1–577.3 eV^40,41^, and 578–578.3 eV for Cr^6+^ (CrO_3_ and/or CrO_4_^2−^)^40^. The Ni signal appears for the 304 samples after sputtering with Ar^+^ (Table 2) and the signals were quite low. XPS peaks are from Ni^2+^ oxide (NiO) 853.6–853.8 eV and Ni^3+^ oxide (Ni_2_O_3_) 854.6–855.6 eV. Sometimes the nickel oxide and nickel oxide hydroxide contents are so small that in some studies they were not even detected in the passive film of stainless steel^42^. According to other publications, Ni^2+^ oxide (NiO) is 854.3 eV^43^, Ni^3+^ is 855.6 eV^44^, and hydroxide Ni(OH)_2_ is 857.9 eV^45^.

**Figure 4:**
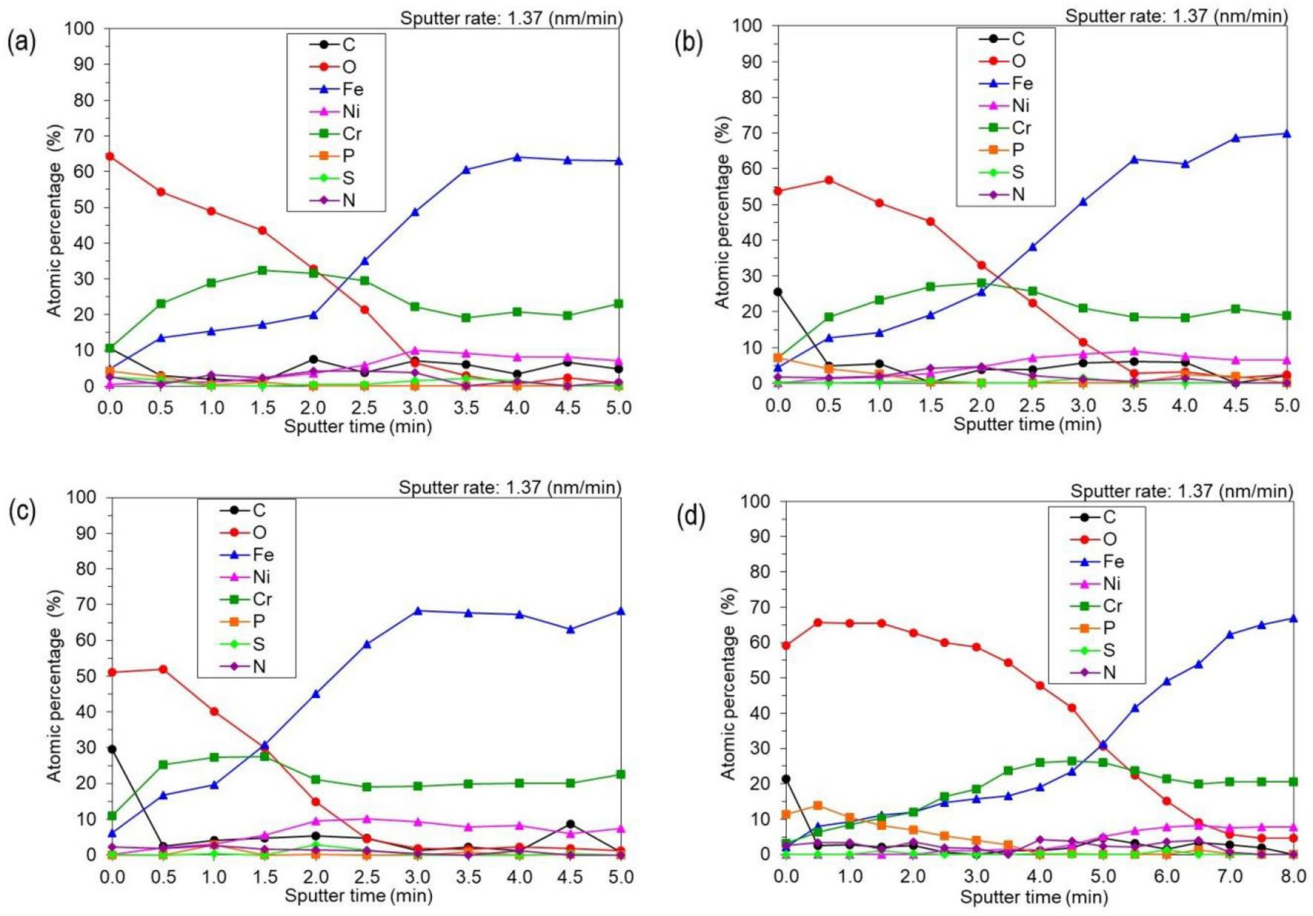
Depth profiles of the surface of samples electropolished in Solutions: (**a**) A_Fe0%_, (**b**) A_Fe6%_, (**c**) B_Fe0%_, (**d**) B_Fe6%_.

Figure 4 shows the atomic depth profiles (in %) of Fe, Cr, Ni, and O in the passive films. The content of Fe is higher than that of Cr. After cleaning the surface with Ar^+^ ions, we received a higher content of chromium and reduced the content of nickel (< 4 nm), while the zone was enriched with nickel content (< 4 nm) and the bulk^46^. After sputtering with Ar^+^, the content of free oxygen was lower than on the top of the surface sample. The procedure of sample cleansing after the electropolishing process, consisting in three stages of rinsing in distilled water and 20 min of cleansing in an ultrasonic bath, did not guarantee the total removal of contaminants (sulphates and phosphates) from the surface of the steel processed in Solution B_Fe-6%_, even though it was sufficient for the other samples.

The changes in the surface composition of stainless steel, which are observed by analyzing the deconvolution and depth profiles, are related to the reactions taking place during the electropolishing process. The components of the steel dissolve on the anode upon which, with the appropriate oxygen and current density, oxygen gas is released simultaneously with hydrogen on the cathode.

2 $${\text{Fe}} \to {\text{Fe}}^{3 + } + 3{\text{e}}^{ - }$$

3 $${\text{Cr}} \to {\text{Cr}}^{3 + } + 3{\text{e}}^{ - }$$

4 $${\text{Ni}} \to {\text{Ni}}^{2 + } + 2{\text{e}}^{ - }$$

5 $$2{\text{H}}_{2} {\text{O}} \to {\text{O}}_{2} + 4{\text{H}}^{ + } + 4{\text{e}}^{ - }$$

6 $$2{\text{H}}_{3} {\text{O}}^{ + } + 2{\text{e}}^{ - } \to 2{\text{H}}_{2} {\text{O}} + {\text{H}}_{2}$$

The process of anodic dissolution of metals and their transition to solution can be represented by the equation:

7 $${\text{M}} + {\text{xH}}_{2} {\text{O}} - z{\text{e}}^{ - } \to {\text{M}}^{{{\text{z}} + }} + {\text{xH}}_{2} {\text{O}}$$

8 $${\text{M}} + {\text{xH}}_{2} {\text{O}} + {\text{yA}}^{ - } - {\text{ze}}^{ - } \to \left[ {{\text{MA}}_{{\text{y}}} } \right]^{{{\text{z}} - {\text{y}}}} \cdot {\text{xH}}_{2} {\text{O}}$$

The mechanism of the electrochemical polishing process has been presented in previous studies^47^. The process of water particles’ diffusion and the migration of anions towards the anode is a result of the simultaneous formation of an oxide layer and a diffusion layer. They enable the simultaneous electrochemical formation and chemical dissolution of the passive oxide layers. Due to the uneven structure of the electropolished surface, the obtained layer has a variable thickness. In the peak areas, the rate of chemical reactions is faster due to the easier access of water particles and anions, which reduces the thickness of the oxide layer, while in the indentations the process is slower, resulting in a thicker layer. The difference in the rate of migration and diffusion of acceptors of the dissolution process springs from the competitiveness of formation and dissolution of the oxides. As a result, even small changes in the rate of these processes have a considerable impact on the dissolution rate of unevenness on the anode surface and its final smoothing. Marcus et al.^48^ used STM images for high resolution surface analysis to investigate at small time and length scales the nucleation and growth mechanisms of the surface oxide on a model stainless steel. The described process takes place in several stages. On the initial oxide-free surface, the process of Cr(III) oxide nucleation takes place. Cr atoms present at each atomic edge are consumed and remoted, leaving behind vacancies in the top layer. Due to the formation of vacancies, the chromium from the lower layers is shifted while regions depleted in Cr are rearranged. This process takes place first at the terrace borders, while away from the step edges delayed Cr(III) oxide nucleation takes place. Iron oxidation can take place in regions where all initially presented chromium is oxidized. This leads to the formation of local centers of iron oxides, which differ in structure and morphology from chromium oxide rich nuclei. The increased amount of chromium in the passive layer has a positive effect on corrosion resistance. Based on the XPS measurements, the total Cr/Fe ratio in the surface film formed, concerning the different compositions of electrochemical polishing baths, has been calculated (Table 3). For samples as received, this value was 0.75–0.86. The highest chromium-to-iron ratio of 1.88 was observed on the sample polished in solution A_Fe0%_. Other samples had a similar level of the Cr/Fe ratio of about 1.4. Oxide film formed on the sample surfaces after EP was characterized by a significantly higher atomic Cr/Fe ratio in comparison to the samples untreated “as received” with naturally-grown passive oxide film (Table 3). A similar increase can be seen in the work of Lee and Lai^49^ who observed that the Cr/Fe ratio increased from 0.97 to 2.58 after EP 316L stainless-steel.

**Table 3:** Depth profiles of the surface’s chemical composition after sputtering the sample surface with Ar^+^ ions at a depth corresponding to the increased chromium content.

Solution	Sputter time	Etch depth	C1s	O1s	Fe2p3	Ni2p3	Cr2p	P2p	S2p	N1s	Sum	Cr/Fe
	(min)	(nm)	at%									
A_Fe0%_	1.5	2.1	1.3	43.5	17.2	2.1	32.4	1.1	0.0	2.4	100.0	**1.88**
A_Fe6%_	1.5	2.1	0.4	45.3	19.2	2.9	27.0	0.2	0.8	4.3	100.0	**1.41**
B_Fe0%_	1.0	1.4	4.0	40.1	19.6	3.0	27.4	2.9	0.3	2.7	100.0	**1.40**
B_Fe6%_	3.5	4.8	1.1	54.3	16.6	1.5	23.8	2.7	0.0	0.0	100.0	**1.43**

### Corrosion test results

Samples for corrosion tests were electropolished in baths in the initial phase of exploitation A_Fe0%_, B_Fe0%_, and after a period of intensive exploitation A_Fe6%_, B_Fe6%_. Process parameters: time 15 min, current density 8 A/dm^2^, and temperature 55 °C. Potentiodynamic tests were conducted in a tri-electrode setup: at a temperature of 25 °C ± 1°, in a solution of 0.1 M NaCl, and at a scanning rate of 1 mV/s.

**Figure 5:**
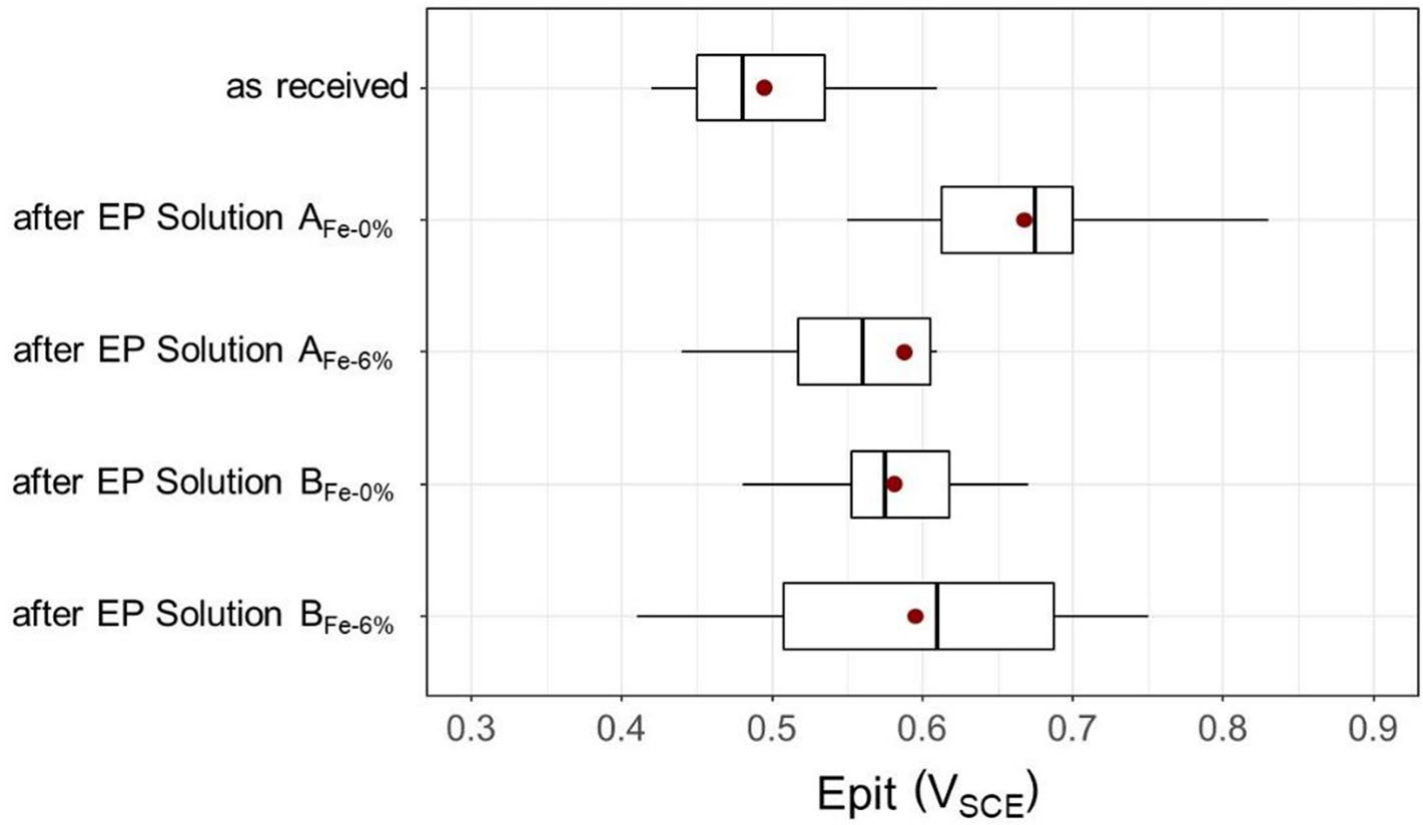
Corrosion tests results obtained in the 0.1 M environment of NaCl solution.

Raw samples were characterised by a lower value of pitting potential (Epit: 0.42–0.55 V_SCE_) than the samples after electrochemical processing (Fig. 5). The highest values of pitting potential Epit were obtained in samples after electropolishing in Solution A_Fe0%_, where the median for ten measurements was 0.68 V_SCE_. The application of the same bath, although after a long-term exploitation Solution A_Fe6%_ led to a deterioration of the results, was manifested in the form of a median of 0.56 V_SCE_ for this series. As far as the bath with an addition of triethanolamine was concerned, similar results were obtained both for the bath at the initial stage (Solution B_Fe0%_) and after a long exploitation (Solution B_Fe6%_). For both the analysed baths, it was noted that for baths after long exploitation, the Epit values within a single series were highly varied, and their range was significantly larger than for samples from the series tested in baths at the initial stage of exploitation. This might confirm the unevenness of surface and potential defects that may emerge in baths with a high concentration of metal (mainly iron) ions. The obtained results allow us to conclude that the process of electropolishing 304 stainless steel samples contributes to the increase in pitting potential and improves resistance to corrosion. The results of the corrosion tests and XPS analyses of the chemical composition of the passive film correspond to those presented in the work mechanism of the passive film’s formation. The lowest Epit median was obtained for the samples as received which were characterized by Cr/Fe ratio 0.86. The best surface quality was obtained for a sample that had been electropolished in Solution A_Fe0%_. The Cr/Fe ratio was 1.88 and the median resistance to the pitting corrosion Epit was 0.67 V_SCE_. The Epit median of samples that had been electropolished in Solution B, both at the initial stage and after long-term exploitation, fell within the range 0.56–0.61 V_SCE_ while their Cr/Fe ratio ranged from 1.43 to 1.40.

In all cases of the analyzed electropolishing solutions, a better composition of the passive layer was obtained than for the as-received sample, which is related to the process mechanism and enrichment of this layer with chromium. Even intensive and long-term exploitation of the process bath in the range of up to 6% Fe, although it significantly worsens the quality of surface smoothing, has a positive effect on its corrosion resistance.

However, the passive layer on the surface of electropolished samples is highly resistant, especially in the case of environments containing chlorides, where it is susceptible to damage. Marcus et al.^50^ have presented a model of passivity breakdown that takes into account the nanostructure of the oxide layer. In the case of electrolyte containing chlorine, Cl^−^ ions compete with OH^−^ ions, which in turn increases the dissolution rate and inhibits the growth of the passive film. This leads to a faster thinning of the passive layer and the depassivation of less resistant places. Taking into account the composition of the passive layer and the results of corrosion resistance, electropolishing of 304 steel in an electrolyte consisting mainly of sulphuric (VI) acid and phosphoric (V) acid allows for better results compared to untreated surfaces. This dependence was observed both in the case of the baths at the initial stage of exploitation without contaminants and after long-term exploitation, when the amount of contaminants increases significantly. Due to the assessment of the obtained surface in terms of the expected visual result of electropolishing, the mathematical model makes it possible to determine the level of bath contamination for which these effects can be achieved, as well as when it may become impossible. However, even using a contaminated electrolyte that resulted in a surface of unsatisfactory gloss and roughness still gave rise to a surface with better corrosion resistance than the untreated sample.

### Mathematical model

The experimental input data concerning roughness and gloss were the basis for creating logistic and square models for Solution A and Solution B, respectively. Detailed characteristics of model parameters are presented in an electronic supplementary in Tables S1 and Tables S2. Then, for the analysed ranges of parameters (i = 4–8 A/dm^2^, T = 35–55 °C, t = 0–45 min, %Fe = 0–5), the results were generated with temperature steps of 1 °C, for 1 min time intervals, current density at a step of 1 A/dm^2^, and contamination at a step of 1% Fe. For each of these points, the models made it possible to calculate the values that determine surface quality, i.e. roughness and gloss, as well as the weight loss per surface area unit. Solution B was characterised by higher values for RMSE and R^2^, respectively for gloss and roughness models, than for Solution A.

Gloss models were prepared based on the logistic function (see electronic Supplementary Table S1). Due to the fact that the results for Solution A differed significantly, the authors decided to divide them into groups corresponding to contamination from 0% to less than 4% and from 4 to 5%, inclusively. This enabled improving the RMSE value in the respective ranges. The model developed for Solution B was based on the whole data range for contamination from 0 to 5%, inclusively. As far as the gloss models were concerned, 28,980 results were generated for each of the baths. For Solution A, the degree of process bath contamination up to the level of 4% Fe had only a marginal influence on the electropolishing results obtained. After this threshold is exceeded, the time required to obtain satisfactory surface gloss results becomes longer. As for temperature, its influence was stronger in the lower ranges of the applied temperatures. For baths at the initial stage of exploitation, with contamination already reaching approx. 2% Fe, the application of either high temperature and low current density or low temperature combined with high current density led to similar results. It was noted that higher current density values are more stable as contamination increases, such that they can bring positive results even if the Fe concentration exceeds 4%. The results of tests on samples electropolished in Solution B demonstrated that the negative influence of contamination on the obtained gloss result increased much faster, which was particularly noticeable in higher temperatures. The application of a temperature of 55 °C and low current density resulted in a significant deterioration of gloss already at a degree of contamination of 1% Fe, while for higher current densities similar results were obtained even at a contamination of 3% Fe.

**Figure 6:**
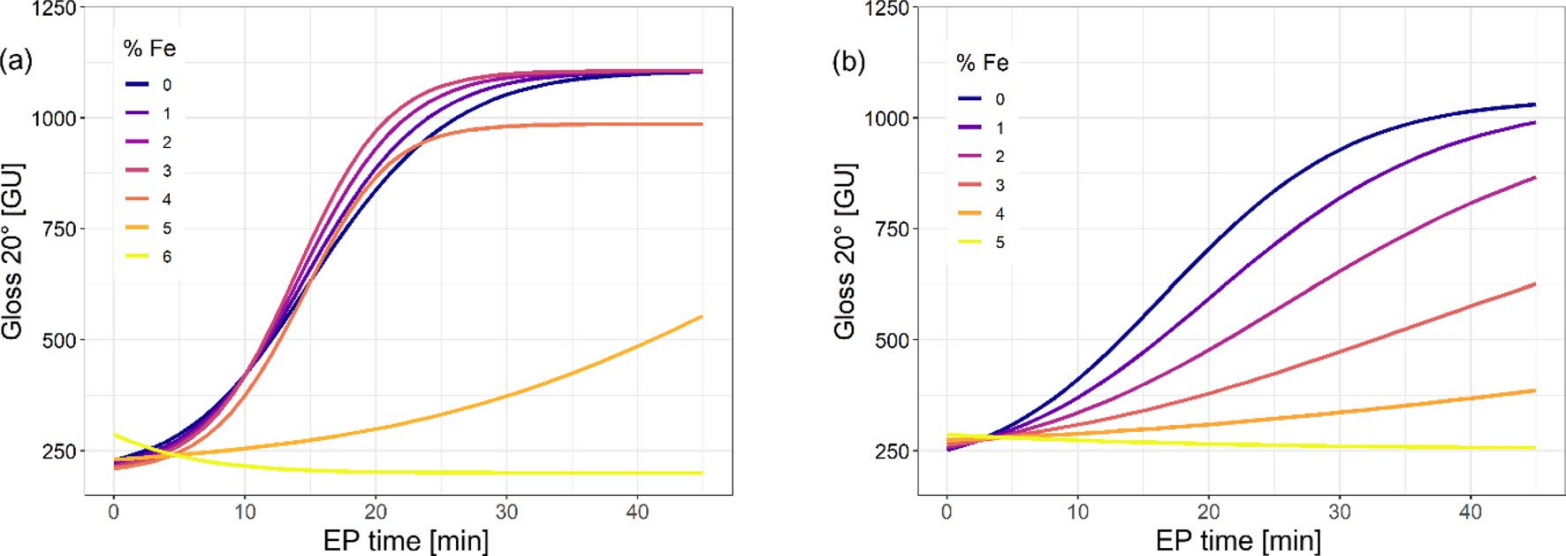
A simplified graphical illustration of the gloss mathematical model: (**a**) Solution A, (**b**) Solution B.

The model takes into account so many variables and dependencies between them that presenting it in a readable graphic form required simplification. A simplified visualization (Fig. 6) of the gloss model, made on the basis of values obtained for the averaged conditions of temperature and current density, makes it possible to observe general trends and differences between the tested solutions. In the case of Solution A, similar results were obtained in the range of 0–3% Fe, before a slight deterioration for 4% Fe was observed, followed by a sudden decrease in the value of the obtained gloss in the final stage of bath exploitation. In the case of Solution B, changes in the obtained gloss values ​​depending on the contamination of the bath are more uniform. There are no sudden jumps, but only a slow decrease in surface quality with the operating time and increasing contamination.

Roughness models were constructed based on the square functions for time. The basis for both models, i.e. for Solution A and Solution B, was the range of contamination data from 0 to 4%, inclusively. As far as roughness is concerned, the variability of test results was relatively high compared to differences caused by the influence of variable process parameters. Due to the specificity of surfaces obtained in the electropolishing process and roughness measurements, even the measurement results of the sample are characterised by a high variability, resulting in a high variance of the roughness model. For the roughness models, 24,150 results were generated for each of the baths. Roughness always decreased with the prolongation of the process regardless of other parameters. This was true for both baths. For Solution A, the current density was more important for the description of the dynamics of changes. For lower current density values, a significant decrease in roughness was achieved after approx. 15 min, while for higher densities the process was visible from the very beginning which started to slow down gradually after approx. 30 min. These changes were more dynamic for higher bath contamination at lower current density. For Solution B, the course of this process was similar except for a combination of low temperature and high current density, wherein a monotonous increase in roughness was noted for the contamination level exceeding 2%. The lowest roughness values were obtained for a combination of high temperature and high current density.

The latter has a decidedly stronger influence on the increase in weight loss than temperature. Additionally, it is strongly linked to contamination. For all the analysed measurement series, the application of low current densities resulted in lower weight loss values. The weight loss increased with growing current density. The lowest weight loss was noted for the combination of low temperatures and low current densities. For higher current densities, at the initial stage of bath exploitation (up to approx. 3% Fe), the contamination did not have a significant influence on changes in the samples’ weight loss. However, as the exploitation time increases, these changes start to grow exponentially, such that at 6% contamination the weight loss was lower by half than the value for the low contamination level.

Table 4 presents ten roughness and gloss results generated by means of the created model for each of the analysed baths. The selected results meet the adopted criterion of the obtained surface’s satisfactory quality (gloss above 800 GU and roughness lower than 140 nm), while at the same time attempting to achieve the lowest weight loss possible. The application of such values in the process of selecting the optimum criteria takes into account both the need to obtain a sufficiently glossy and smooth surface and the need to minimise the contamination of the bath with metal ions. For Solution A, the main factor that determined the selection of parameters was weight loss. Due to the fact that gloss and roughness results were similar and satisfactory in the bath contamination range from 0 to 4%, meeting the boundary conditions (Ra < 140 nm, gloss > 800 GU) posed no real challenge. As the process of anodic dissolution slowed down with an increasing bath contamination, in the next stage which considered the need to maintain the lowest weight loss among the obtained results, all the selected values corresponded to a 4% contamination. As for Solution B with good gloss and roughness results in the contamination range from 0 to 2% Fe, the operational efficiency of the bath deteriorated. The obtained results worsened as contamination increased. As a result, the key element in choosing the optimum parameters for Solution B was the need to meet the adopted boundary conditions. The generated sets of optimum parameters fluctuated within the low temperature range 35–37 °C and highest analysed current density of 8 A/dm^2^. Ensuring a sufficiently high gloss and low roughness in these cases would be possible using baths at the initial stage of exploitation 0–1% Fe, while the samples’ low weight loss may be achieved by shortening the duration of the process. The suggested optimum electropolishing time was 16–19 min. Another parameter worth considering when selecting the optimum parameters is the electric load per unit expressed in A min/cm^2^. This parameter directly influences the energy consumption of the process. Minimising the A min/cm^2^ value allows for reducing the power consumption, which is an important aspect in terms of environmental protection.

**Table 4:** List of the best results that meet the set objectives (Ra < 140 nm, gloss > 800 GU, Δm tending to a minimum value) generated based on the models.

Bath	Bath contamin.	T	t	i	Δm	(Δmmin − Δm)/Δmmin	Q	(Q_min_ − Q)/Q_min_	Gloss 20°	Gloss lower	Gloss upper	Ra	Ra lower	Ra upper
	%Fe	°C	min	A/dm^2^	mg/dm^2^	%	A min/cm^2^	%	GU	GU	GU	nm	nm	nm
Solution A	4	37	16	8	511	0.0	1.28	− 6.7	802	684	919	132	90	174
Solution A	4	38	16	8	519	− 1.6	1.28	− 6.7	807	689	925	132	90	174
Solution A	4	35	17	8	526	− 3.2	1.36	− 13.3	833	714	950	130	88	173
Solution A	4	39	16	8	527	− 3.3	1.28	− 6.7	813	695	929	132	90	174
Solution A	4	35	19	7	530	− 3.9	1.33	− 10.8	854	735	972	139	97	181
Solution A	4	40	16	8	535	− 4.9	1.28	− 6.7	818	700	935	132	90	174
Solution A	4	36	17	8	535	− 4.9	1.36	− 13.3	838	719	955	130	88	173
Solution A	4	36	19	7	538	− 5.6	1.33	− 10.8	858	739	975	139	97	181
Solution A	4	45	15	8	539	− 5.7	1.20	0.0	802	684	919	134	92	176
Solution A	4	41	16	8	543	− 6.5	1.28	− 6.7	823	705	940	132	90	174
Solution B	0	35	16	8	612	0.0	1.28	0.0	836	679	990	129	71	187
Solution B	1	35	18	8	619	− 1.1	1.44	− 12.5	807	651	960	128	70	186
Solution B	0	36	16	8	621	− 1.4	1.28	0.0	832	676	987	133	76	191
Solution B	0	37	16	8	629	− 2.8	1.28	0.0	829	672	983	138	80	196
Solution B	0	35	17	8	649	− 5.9	1.36	− 6.3	868	712	1022	128	70	186
Solution B	1	35	19	8	652	− 6.5	1.52	− 18.8	836	680	989	126	68	184
Solution B	0	36	17	8	658	− 7.4	1.36	− 6.3	864	708	1018	132	74	190
Solution B	1	36	19	8	661	− 8.0	1.52	− 18.8	828	673	982	131	73	189
Solution B	0	37	17	8	666	− 8.8	1.36	− 6.3	861	705	1014	137	79	195
Solution B	1	37	19	8	671	− 9.5	1.52	− 18.8	820	665	974	135	77	193

## Conclusion

Based on laboratory tests of electropolishing steel in variable time, temperature, current density and bath contamination conditions, the authors have created a multi-factorial mathematical model. The ranges of analysed parameters were selected based on the specificity of the process in industrial conditions and related limitations, e.g. the possibility to apply only low current densities due to the fairly large surface area of electropolished details.

The presented mathematical model enables generating results within the range selected by the user. Depending on the expected result and requirements, the model also makes it possible to select the values of parameters that influence them. As such, the authors have selected the values of parameters that allowed them to achieve satisfactory surface quality (roughness Ra < 0.14 and gloss above 800 GU), while at the same time maintaining the lowest weight loss possible. Minimising the weight loss of electropolished details is an important issue related to the electropolishing process, as it directly influences the amount of contaminants that emerge during the process. These conditions were fulfilled for both Solution A and Solution B when high current density of 8 A/dm^2^ and low temperatures in the range of approx. 35–40 °C were used. The duration of the process necessary to obtain satisfactory results in all cases ranged from 15 to 19 min. However, the selected ranges of bath contamination differed significantly. For Solution A, boundary conditions were met by individual values throughout the analysed contamination range, but those with the lowest weight loss fell within the range of 3–4% Fe. For Solution B, points that met the boundary conditions were observed in the bath contamination range of 0–3% Fe, whereas those with the lowest weight loss fell within the range of 0–1% Fe. This difference stems from the diverse working characteristics of the applied baths during their exploitation.

XPS analyses of samples electropolished in Solution A and Solution B were conducted both at the initial and final stage of exploitation. The highest value of the Fe/Cr ratio (1.88) was noted for Solution A_Fe=0%_. These results were also reflected in the pitting corrosion resistance tests, where the application of Solution A_Fe=0%_ resulted in the highest median values of Epit = 0.69 V_SCE_ among all the analysed samples. The other variants were characterised by similar Fe/Cr ratios of approx. 1.4 V_SCE_ and a median Epit value in the range of 0.56–0.61 V_SCE_.

## Supplementary Information

Supplementary Tables.
