# Supplementary material for: A multi-factorial mathematical model for the selection of electropolishing parameters with a view to reducing the environmental impact
Source: Sci Rep. 2021 May 3;11:9443. doi: 10.1038/s41598-021-88731-5 (PMC8093221; doi:10.1038/s41598-021-88731-5)
Supplement: Supplementary file 1 — Supplementary Tables. [file 41598_2021_88731_MOESM1_ESM.docx]

Supplementary Material for **A multi-factorial mathematical model for the selection of electropolishing parameters with a view to reducing the environmental impact**

Paweł Lochyński**^*^**^,1^, Sylwia Charazińska^1^, Maciej Karczewski^2^ & Edyta Łyczkowska-Widłak^1^

^1^ Wrocław University of Environmental and Life Sciences, Institute of Environmental Engineering, Grunwaldzki Square 24, 50-363, Wrocław, Poland

^2^ Wrocław University of Environmental and Life Sciences, Department of Mathematics, Grunwaldzka 53, 50-357 Wrocław, Poland

Table S1. Characteristics of the parameters for logistic models for Solution A and Solution B

|  | **Solution A** | | | | | | **Solution B** | | |
| --- | --- | --- | --- | --- | --- | --- | --- | --- | --- |
|  | **Fe < 4%** | | | **Fe ≥ 4%** | | | **Fe ≤ 5%** | | |
| **term** | **estimate** | **std.error** | **p.value** | **estimate** | **std.error** | **p.value** | **estimate** | **std.error** | **p.value** |
| α | 906.3 | 4.574 | <0.0001 | 786.12 | 11.76 | <0.0001 | 829.69 | 9.754 | <0.0001 |
| Β | -10.95 | 0.7085 | <0.0001 | -24.52 | 2.448 | <0.0001 | -11.53 | 0.71 | <0.0001 |
| 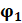- time | 0.03606 | 0.0258 | 0.1627 | 1.42 | 0.112 | <0.0001 | 0.249 | 0.014 | <0.0001 |
| 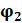– current density | 0.8883 | 0.0795 | <0.0001 | 0.644 | 0.047 | <0.0001 | 1.128 | 0.086 | <0.0001 |
| 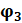– temperature | 0.09067 | 0.0142 | <0.0001 | 0.253 | 0.0373 | <0.0001 | 0.137 | 0.013 | <0.0001 |
| 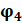– contamination | 0.7281 | 0.1337 | <0.0001 | 3.47 | 0.507 | <0.0001 | 1.174 | 0.146 | <0.0001 |
| 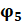– time * current density | -0.01176 | 0.0025 | <0.0001 | -0.0106 | 0.00142 | <0.0001 | 0.006 | 0.001 | <0.0001 |
| 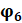– time * temperature | 0.006228 | 0.00065 | <0.0001 | -0.00046 | 0.00027 | 0.091 | -0.003 | 0.0002 | <0.0001 |
| 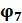– time * contamination | 0.02712 | 0.0055 | <0.0001 | --0.253 | 0.0215 | <0.0001 | -0.039 | 0.0018 | <0.0001 |
| 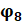– temperature * contamination | -0.02177 | 0.0022 | <0.0001 | -0.0519 | 0.0087 | <0.0001 | -0.012 | 0.0023 | <0.0001 |
| 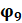– current density * temperature | -0.00844 | 0.0013 | <0.0001 | - | - | - | -0.015 | 0.0016 | <0.0001 |
| 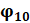– current density * contamination | - | - | - | - | - | - | 0.112 | 0.011 | <0.0001 |
| RMSE | 51.56 | | | 58.46 | | | 75.39 | | |

Table S2. Characteristics of the parameters of square models for Solution A and Solution B

|  | **Solution A** | | | **Solution B** | | |
| --- | --- | --- | --- | --- | --- | --- |
|  | **Fe ≤ 4%** | | | **Fe ≤ 4%** | | |
| **term** | **estimate** | **std.error** | **p.value** | **estimate** | **std.error** | **p.value** |
| intercept | 0.165 | 0.00601 | <0.0001 | 0.073 | 0.0149 | <0.0001 |
| time | -0.249 | 0.122 | 0.0424 | -0.492 | 0.169 | 0.00362 |
| time^2^ | -0.822 | 0.122 | <0.0001 | -0.0519 | 0.168 | 0.758 |
| current density | -0.0006 | 0.00052 | 0.252 | 0.00114 | 0.00221 | 0.605 |
| temperature | -0.00031 | 0.00011 | 0.0064 | 0.00374 | 0.000316 | <0.0001 |
| contamination | 0.000775 | 0.0025 | 0.761 | 0.00056 | 0.0035 | 0.873 |
| time * current density | -0.0585 | 0.0106 | <0.0001 | -0.243 | 0.0147 | <0.0001 |
| time^2^ * current density | 0.113 | 0.0106 | <0.0001 | 0.193 | 0.0146 | <0.0001 |
| time * temperature | 0.00274 | 0.0022 | 0.216 | 0.047 | 0.00305 | <0.0001 |
| time^2^ * temperature | 0.00461 | 0.00219 | 0.035 | -0.0431 | 0.00301 | <0.0001 |
| time * contamination | -0.0286 | 0.0144 | 0.047 | 0.0457 | 0.0198 | 0.0213 |
| time^2^ * contamination | -0.052 | 0.0146 | 0.00037 | 0.092 | 0.0201 | <0.0001 |
| current density * temperature | - | - | - | -0.00027 | 0.000046 | <0.0001 |
| current density * contamination | -0.00092 | 0.000225 | <0.0001 | 0.00192 | 0.000309 | <0.0001 |
| temperature * contamination | 0.000141 | 0.000047 | 0.0029 | -0.00028 | 0.000065 | <0.0001 |
| R^2^ | 46.15% | | | 67.93% | | |
